# Barriers and Facilitators to Implementing Digital Health Technologies for Remote Management of NCDs in Rural Areas: Mixed Methods Systematic Review

**DOI:** 10.2196/93875

**Published:** 2026-07-31

**Authors:** Serine Sahakyan, Selai Akseer, Lusine Abrahamyan, Olivia Metcalf, Sara Allin, Richard Chenhall, Emily Seto

**Affiliations:** 1 Institute of Health Policy, Management and Evaluation, Dalla Lana School of Public Health, University of Toronto, 4th Floor, 155 College St, Toronto, ON, M5T 3M6, Canada, 437 838 8414; 2 Melbourne School of Population and Global Health, Faculty of Medicine, Health and Dental Science, The University of Melbourne, Melbourne, Victoria, Australia; 3 Centre for Digital Transformation of Health, The University of Melbourne, Melbourne, Victoria, Australia; 4 Centre for Digital Therapeutics, University Health Network, Toronto, ON, Canada

**Keywords:** digital health, rural population, noncommunicable diseases, mHealth, eHealth, telemedicine, remote patient monitoring, CFIR, chronic disease management, rural health services, mixed methods, Implementation

## Abstract

**Background:**

Digital health technologies (DHTs) have the potential to improve care delivery and outcomes for patients with noncommunicable diseases. Yet their implementation in rural settings remains uneven, and the factors influencing uptake are not well understood.

**Objective:**

This mixed methods systematic review aimed to identify barriers and facilitators influencing the implementation and use of DHTs for remote management of noncommunicable diseases in rural areas.

**Methods:**

We searched Medline, Embase, and CINAHL from inception to February 12, 2026, using terms related to digital health, noncommunicable diseases, and rural settings. Following the Joanna Briggs Institute methodology for mixed-method systematic review, we synthesized quantitative and qualitative studies. Barriers and facilitators were categorized using the Consolidated Framework for Implementation Research, and study quality was appraised using the Mixed Methods Appraisal Tool.

**Results:**

From the initial 1491 records, 14 studies met the inclusion criteria, with most conducted in high-income countries (n=11). Key barriers included technical challenges (software instability and hardware issues), poor internet connectivity, financial constraints, and workforce constraints, such as staff shortages and heavy workloads. Key facilitators included user-friendly technology design, strong leadership, effective teamwork, and ongoing communication. Evidence was predominantly qualitative, with only limited quantitative data available.

**Conclusions:**

DHTs show promise for improving access and continuity of care for cardiovascular disease, hypertension, and diabetes in rural settings; however, their impact is constrained by structural inequities, including limited broadband access, workforce shortages, and financial fragility. These findings highlight important implications for research, policy, and practice, including the need for rigorous mixed methods evaluations sensitive to rural contexts, long-term equity-oriented financing mechanisms, and strengthened organizational readiness to support effective DHT uptake.

## Introduction

Noncommunicable diseases (NCDs) continue to be one of the top public health issues, accounting for more than 70% of causes of deaths worldwide [1,2]. According to the World Health Organization, the majority of NCD deaths are due to cardiovascular diseases (CVD) (17.9 million people annually), followed by cancers (9.3 million), chronic respiratory diseases (4.1 million), and diabetes (2.0 million, including kidney disease deaths caused by diabetes) [2]. NCDs impact both high- and low-income countries [2,3]; however, their burden is disproportionately borne by populations in low-resource settings and rural areas [1,2]. Rural populations often access specialized care, as well as prevention, timely diagnosis, and treatment of NCDs [4,5]. This lack of access leads to poorer health outcomes and higher mortality rates [2,6,7]. Furthermore, failure to properly manage the prevalent NCDs negatively affects human health and a country’s economy, increasing premature mortality and reducing workforce productivity [8,9].

Digital health technologies (DHT) (ie, mobile apps, telemedicine, and telemonitoring) [10] can improve health care delivery and disease management of patients with NCDs [11]. These technologies, often delivered through digital platforms or mobile apps, can provide remote access to evidence-based interventions, clinical support, and monitoring tools for patients with NCDs. Chronic NCDs such as CVDs, hypertension, and diabetes, which require lifelong monitoring and patient self-management, share common challenges that DHTs can address [12,13]. In rural areas where access to health care facilities and specialists may be limited, DHTs can bridge gaps in care by enabling remote monitoring and management of NCDs [14]. In addition, they can have significant positive impacts on disease self-management and health-related behaviors, including improved adherence to treatment and medication intake [11,13,15].

Rural regions often experience limited health care workforce capacity, greater travel distances, and reduced access to specialist care [16]. In addition, infrastructure limitations, such as unstable internet connectivity, and lower availability of technical support and digital resources may constrain the implementation and sustained use of DHTs [17]. Rural populations may also differ in socioeconomic characteristics, digital literacy, and device access, all of which can affect engagement with DHTs [18]. These contextual differences suggest that implementation barriers and facilitators identified in urban settings may not be directly transferable to rural health care environments [19].

In this review, “remote management” refers to the broader application of DHTs to support clinical care and self-management, while many included interventions primarily involve remote patient monitoring functions.

The demonstrated effectiveness, safety, and feasibility of DHTs position them for broader national and international impact beyond their country of origin. However, uptake and implementation face sustained challenges. The adoption of DHTs involves two core processes: uptake and implementation [20]. Uptake refers to the willingness of users, whether individuals, organizations, or communities, to adopt and integrate digital technologies into their practices or daily lives [21]. It assesses the extent to which users embrace and use the technology [21]. Meanwhile, implementation involves the process of putting DHTs into practice or integrating them into existing systems, processes, or workflows [22]. Various factors, such as organizational readiness, stakeholder engagement, infrastructure, and training, are considered to facilitate the successful uptake and integration of the technology [23]. However, numerous challenges hinder the uptake and sustained implementation of DHTs. Some countries struggle with system-level integration and ensuring the interoperability of DHTs [24], while others face difficulties related to user-friendly designs [25], and encounter disparities in usage among different population groups [26].

Although previous systematic reviews have explored implementation factors for DHTs, none have explicitly focused on their use in the remote management of NCDs in rural settings [27,28]. This gap highlights the need for a focused synthesis to inform the development and implementation of effective digital health solutions in underserved communities.

This systematic review aims to identify and synthesize the key barriers and facilitators influencing the implementation and uptake of DHTs for remote management of NCDs in rural areas, guided by the Consolidated Framework for Implementation Research (CFIR). Specifically, this review addresses the following research question: what are the barriers and facilitators influencing the implementation and uptake of DHTs for remote management (both clinical and self-management) of NCDs in rural areas?

## Methods

### Overview

This study protocol was developed and reported in accordance with the PRISMA-P (Preferred Reporting Items for Systematic Reviews and Meta-Analysis Protocols) checklist and reporting guideline (Checklist 1) [29].

### Eligibility Criteria

This systematic review included original, peer-reviewed studies that examined the implementation or uptake process of DHTs. These included mobile health solutions, such as smartphone apps and multimedia message services, wearable devices, and telemedicine options such as videoconferences and telemonitoring [10] for the remote management of adult patients (18 years and older) with NCDs. The NCDs included in this review were CVDs (eg, heart attacks, stroke, and heart failure), hypertension, and diabetes, as these represent highly prevalent chronic conditions globally [2]. Rural settings were defined based on the classifications or descriptions provided in the original studies. Studies were included only if they explicitly described their setting as rural, remote, nonurban, or underserved. During screening, full-text articles were carefully reviewed to ensure that the context aligned with these definitions. Studies referring to urban settings, or where the context was unclear or insufficiently described, were excluded, as it was not feasible to independently classify the setting.

Observational, experimental, qualitative, and mixed methods studies were considered if they measured, observed, or explored the implementation and uptake processes and related factors. Only articles published in the English language were included. If studies included mixed children and adult patient populations, they were considered for the review if separate findings were reported for adults. Original studies that focused on DHTs exclusively for disease screening, diagnosis, or patient education, or those conducted solely in urban settings, or without specifying the geographic area, were excluded from the study. If the studies included both urban and rural areas and presented separate results, only the findings related to rural areas were considered. No restrictions were applied to year of publication, country of publication, study setting, or adult population age. Case reports, reviews, letters to editors, and conference abstracts were not included in the review.

### Information Sources and Search Strategy

To identify potentially relevant articles, we searched the following bibliographic databases from inception to February 12, 2026: Medline, Embase, and CINAHL. The search strategy used three groups of keywords in the search syntax. It required at least one keyword from each group: (1) digital health–related keywords, which included terminologies that describe subsets of digital health, such as “telehealth,” “mobile health,” “digital therapeutics,” (2) NCD-related keywords, which included general terms, such as “cardiovascular disease” or “chronic diseases,” and specific conditions, such as “diabetes,” “stroke,” and “heart failure,” and (3) rural area-related, such as “rural,” “remote” or “non-urban.”

The search strategy was developed and executed by a professional librarian (EU) and customized specific search syntax based on the indexing requirements of each database, including Medline, Embase, and CINAHL. Full search strategies for all databases are provided in Multimedia Appendix 1.

Two trained researchers (SS and SA) conducted two rounds of independent screening of each article: initially by title and abstract, then by full-text review, according to the predefined inclusion and exclusion criteria. The study selection process is illustrated in the PRISMA (Preferred Reporting Items for Systematic Reviews and Meta-Analysis) flow diagram. Discrepancies between the reviewers were resolved through group discussions until a consensus was reached. Covidence (Veritas Health Innovation) was used to manage the literature database.

### Quality Appraisal Process

Methodological quality was appraised using the Mixed Methods Appraisal Tool (MMAT), version 2018 [30]. The evaluation covered five study designs: qualitative research, randomized controlled trials, nonrandomized studies, quantitative descriptive studies, and mixed methods studies [30]. Two MMAT screening questions were applied to all studies: (1) whether the study had a clear research question, and (2) whether the data collected were appropriate for addressing that question [30]. After this step, design-specific criteria were applied. Consistent with MMAT guidance, we reported criterion-level ratings (ie, “Yes,” “No,” or “Can’t tell”) and summarized the proportion of criteria met within each MMAT domain (ie, each study design category).

### Data Extraction

Data were extracted using a structured form that included several categories: (1) basic information about each study, such as the title, year of publication, author’s name, study design, study participants, and country; (2) details regarding the type of DHT, the NCD condition, and the target population for the DHT; and (3) barriers and facilitators of DHT, which were identified and categorized based on the CFIR [31]. The CFIR provides a systematic way to examine what helps or hinders implementation by organizing findings into five domains: innovation, outer setting, inner setting, individuals, and implementation process [32]. The same two researchers (SS and SA) conducted independent data extraction based on the structured forms, then compared and checked for discrepancies. The complete data extraction sheet is provided in Multimedia Appendix 2

### Analysis

To identify barriers and facilitators influencing the implementation and uptake of DHT for remote management of NCDs in rural areas, the analysis followed the Joanna Briggs Institute methodological approach for conducting a mixed methods systematic review [33]. This review specifically used the convergent segregated approach of the Joanna Briggs Institute mixed methods systematic review methodology. Quantitative and qualitative data were simultaneously and independently analyzed, resulting in the generation of quantitative and qualitative evidence [33].

The quantitative data were synthesized using both descriptive and narrative analyses. For qualitative synthesis, we used a directed content analysis approach. Initially, we categorized the themes according to the five domains of the CFIR framework. Using an inductive method guided by the data, we then described the barriers and facilitators within each theme. Additionally, we examined links between factors across different themes.

During the final integration stage, we examined the quantitative and qualitative findings to identify convergence and divergence (ie, whether evidence from both components aligned or contradicted each other). We also explored which aspects of the qualitative evidence were addressed or overlooked in the quantitative data, and vice versa. Finally, we assessed whether qualitative evidence could elucidate why certain factors were or were not associated with DHT implementation, as indicated by the quantitative evidence.

## Results

### Overview

**Figure 1.**
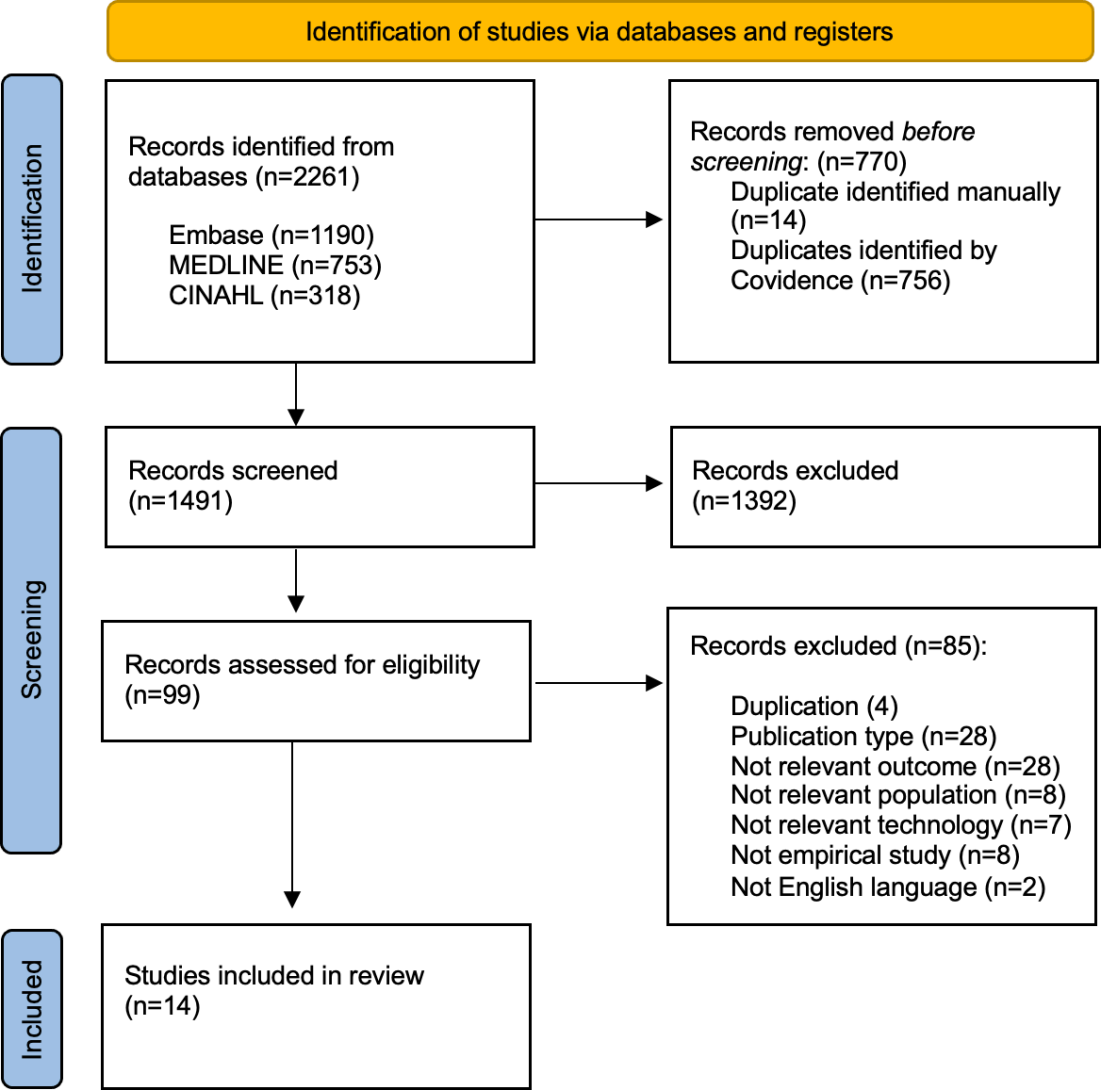
Flowchart for study search and screening.

We identified a total of 2261 records across three databases (Figure 1). After removing 770 duplicates, we screened 1491 titles and abstracts, leaving 99 records for further review. During the full-text screening, 85 records were excluded. Reasons for exclusion included publication type (eg, conference abstracts, gray literature; n=28), having irrelevant outcomes (n=28), irrelevant population (n=8), irrelevant technology (n=7), and nonempirical studies (n=8), and other reasons. A total of 14 studies were included in the final analysis.

**Figure 2.**
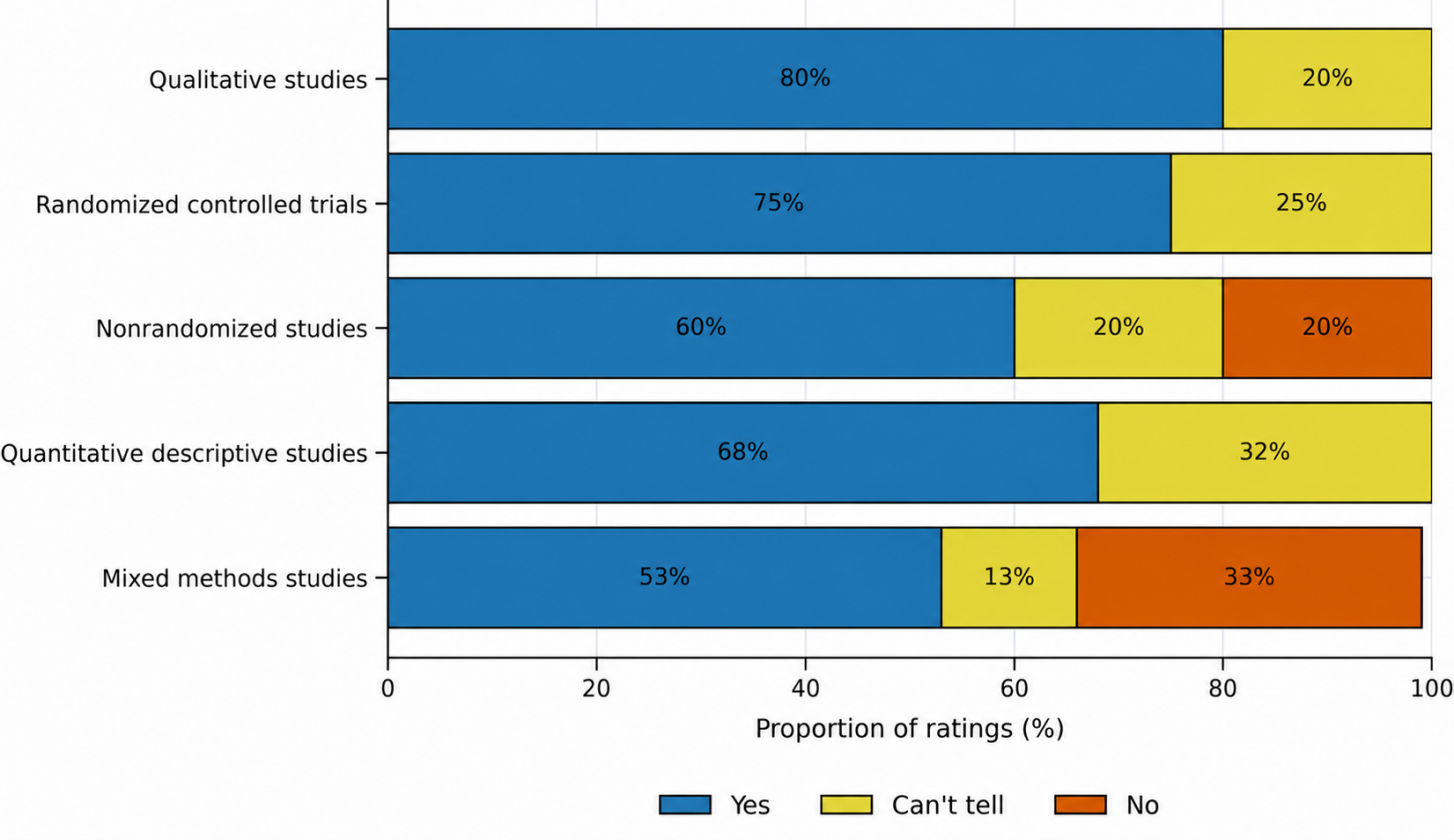
Quality assessment results by study design using Mixed Methods Appraisal Tool (MMAT, 2018): proportions of “Yes,” “No,” and “Can’t tell” ratings.

### Characteristics of Included Studies

Three main design types were used: mixed methods studies (n=7), qualitative studies (n=6), and 1 quantitative study (Table 1). Most studies were conducted in high-income countries such as the United States (n=8) and Australia (n=3). Only 1 study each originated from China, Canada, and Kenya.

**Table 1.** Study characteristics.

Author, published year	Country	Study design	Study population	Study participants, n	NCD^a^ condition and target population	Target rural setting	Digital health technology
Cherry et al (2015) [34]	United States	Mixed methods	Patients and their caregivers	10	Stroke	Community	Wearable devices
Gong et al (2021) [35]	China	Mixed methods	Health care providers, patients, and other stakeholders	50	Stroke	Villages	Mobile health apps
Greer and Abel (2022) [36]	United States	Mixed methods	Patients	30	Hypertension	Community	Smartphone
James et al (2016) [37]	Australia	Qualitative	Health care providers	213	Type 1 diabetes	Unknown	Other technologies (continuous subcutaneous insulin infusion)
Jeffrey et al (2019) [38]	Australia	Qualitative	Patients	30	Type 2 diabetes	Community	Mobile health apps
Jones et al (2023) [39]	United States	Mixed methods	Patients	12	Hypertension	Nurse call center	Remote monitoring devices
Kirkland et al (2023) [40]	United States	Mixed methods	Health care providers	27	Diabetes (type is not specified)	Outpatient clinics	Remote monitoring devices
Kobe et al (2020) [41]	United States	Mixed methods	Patients	230	Type 2 diabetes	Communities	Telehealth and telemedicine
Li et al (2022) [42]	United States	Quantitative	Patients	15	Diabetes (type is not specified)	Outpatient clinics	Other technologies (combination of app, video or phone calls)
Livet et al (2021) [43]	United States	Mixed methods	Implementation stakeholders and health care providers	22	Diabetes (type is not specified)	Clinics	Telehealth and telemedicine
Moloczij et al (2015) [44]	Australia	Qualitative	Health care providers	24	Stroke	Rural hospital	Telehealth and telemedicine
Newell et al (2017) [45]	Canada	Qualitative	—^b^	36	Cardiovascular diseases (the type is not specified)	Medical facilities (not specified)	Telehealth and telemedicine
Peng et al (2016) [46]	United States	Qualitative	Patients	18	Type 2 diabetes	Communities	Mobile health app
Vedanthan et al (2015) [47]	Kenya	Qualitative	Health care providers	12	Hypertension	Rural clinic	Mobile health app

a NCD: noncommunicable diseases.

b Not applicable.

The majority of studies investigated factors influencing implementation and uptake from the perspectives of patients (Table 1). Specifically, six out of 14 studies focused exclusively on patients, while four studies targeted health care providers only (physicians, nurses, and educators). In addition, three studies extended beyond patients and health care providers to include caregivers and implementation stakeholders. One study did not include study participants, consistent with its study design.

### DHT Characteristics

In total, we identified five categories of DHTs across the 14 selected articles (Table 1). These included four mobile apps (some of which allowed providers to communicate with patients through the app, while others did not), 4 telehealth or telemedicine platforms (primarily videoconferencing tools used for remote consultations), a smartphone-based tool, 3 remote monitoring devices, including a wearable device, and other types of DHTs. These technologies in the selected studies were primarily used for monitoring and/or managing NCD conditions in rural areas, such as stroke (n=3), diabetes (n=7), hypertension (n=3), and one unidentified CVD.

### CFIR Domains and Objectives of Included Studies

Table 2 shows that most studies lacked quantitative measurements of factors influencing DHT uptake. Even those using mixed methods approaches, the emphasis was predominantly on qualitative data, with limited use of quantitative metrics to assess barriers and facilitators. Of the 8 studies out of 14 selected, findings regarding factors in the ‘Technology/ innovation’ domain of the CFIR framework were presented using both quantitative and qualitative methodologies. The majority of selected studies investigated factors related to DHT implementation and uptake within the “Inner setting” domain of the framework, followed by the “Individuals” domain. Only a few studies delved into factors related to the “implementation process” domain of the framework, and among the 14 selected studies, only one reported facilitators within this domain.

**Table 2.** Summary of digital health technologies implementation and uptake factors by Consolidated Framework for Implementation Research domains, study type, and study numbers in references.

Framework domains	Implementation		Uptake	
	Qualitatively explored	Quantitatively explored	Qualitatively explored	Quantitatively explored
Technology and innovation	Gong et al [35], Kobe et al [41]	Kobe et al [41], Li et al [42].	Cherry et al [34], James et al [37], Jeffrey et al [38], D Jones et al [39].	—^a^
Outer setting	[47] Gong et al [35], Kirkland et al [40], Vedanthan R [47].	Kirkland et al [40].	Gong et al [35], James et al [37], Jeffrey et al [38], Newell et al [45].	—
Inner setting	Gong et al [35], Kirkland et al [40], Livet et al [43], Newell et al [45], Vedanthan R [47].	Kirkland et al [40].	Gong et al [35], James et al [37], Jeffrey et al [38], Moloczij et al [44], Peng et al [46].	—
Individuals	Gong et al [35], Kirkland et al [40], Vedanthan R [47].	Li et al [42]	Cherry et al [34], James et al [37], Jeffrey et al [38], D Jones et al [39], Peng et al [46].	Greer et al [36]
Implementation process	Kobe et al [41]. Newell et al [45].	Kirkland et al [40], Kobe et al [41]	James et al [37].	—

a Not available.

### Quality Appraisal Findings

Across study types, qualitative studies and randomized controlled trials scored highest, meeting over 70%‐80% of applicable criteria, while nonrandomized and mixed methods studies showed more limitations, with only 53%‐60% of criteria met and higher proportions of ‘No” ratings. Common factors compromising quality, particularly in quantitative studies and the quantitative components of mixed methods studies, included small sample size, unclear representativeness of the target population, insufficient detail on recruitment strategies, and lack of rationale for using mixed methods design or effective integration of both components. Figure 2 provides a visual summary of criterion-level ratings across MMAT study types, illustrating the proportion of “Yes,” “No,” and “Can’t tell” responses within each category.

Additionally, nearly half of the studies (6 out of 14) did not use a framework for designing and implementing their research, while one study used a framework solely for analysis purposes, and the remaining seven studies incorporated different frameworks into their methodologies.

### Main Findings: Factors Influencing DHT Uptake and Implementation

#### Technology Innovation

##### Technical and Application Functionality Issues (barriers)

Technical and application functionality issues, along with user interface challenges, were frequently reported as barriers to successful DHT implementation and uptake [38,42,44,47]. Specifically, hardware and software problems, difficulty in navigation, and readability concerns (eg, font size) hindered the intended usage of the application or device [38,42,44,47]. Additionally, 2 studies reported high device or application costs as barriers [37,38]. Other challenges included a lack of personalized or individualized content [35], and additional barriers are detailed in Textbox 1 .

Textbox 1. **Identified barriers and facilitators influencing the implementation and uptake of digital health technologies for remote management of noncommunicable diseases in rural areas, organized by the Consolidated Framework for Implementation Research framework.**

**Barriers**

 **Technology and Innovation**

- Limited device ownership and infrequent use [35].
- Lack of personalized or individualized content [35].
- Lack of flexibility and adaptability of the app [37].
- Inadequate adaptation to users’ disabilities, such as voice messages not being accessible for individuals with hearing impairments [35].
- Health care providers’ difficulty in keeping up with technological advances [37].
- High device or application cost [37,38].
- Size and placement challenges of the device [34].
- Usability, wearability, and device adjustment difficulties [34,42].
- Lack of trust in the device accuracy, reliability, and usability concerns from patients’ and providers’ perspectives [39,44].
- Technical and application functionality issues: hardware and software problems, connectivity issues, and other functionality problems affecting the intended usage of the application or device [38,42,44,47].
- User interface challenges: including difficulty in navigation and readability due to font size [38,47].

 **Outer setting**

- Implementation cost [35].
- Insufficient funding for human resources maintenance [37,45].
- Inadequate provider reimbursements, coverage for digital health technology (DHT) services (eg, telehealth visits) [37,45].
- Patients limited access to technology and sustainable internet connectivity [35,37,38,40].

 **Inner setting**

- Preexisting heavy workload and competing programs [35,47].
- Limited staffing capacity due to a small workforce and high turnover rates [37,40].
- Lack of staff skilled in the technology [37].
- Lack of space, supplies, and infrastructure within the implementing clinics [40,44].
- Insufficient encouragement or enforcement by health care providers for patient utilization of DHTs [38,46].
- Administrative issues [47].
- Lack of IT expertise [47].

 **Individuals**

- Patients’ low awareness, adherence, or cooperation [35].
- Poor health care providers’ and patients’ technology literacy and self-perception [35,38,46].
- Health care providers’ low educational attainment and language barriers [40].
- Health care providers and patients with low literacy [38,40].
- Health care providers’ confidence and value [44].
- Health care providers and patients lacking needs and motivation [34,42].
- Patient-related factors affecting adherence and engagement, including older age, forgetfulness, time constraints, and major life events [36,42].
- Resistance from traditionalist health care specialists to technological adoption [37].
- Reluctance of patients to take responsibility for their own health [46].
- Patients’ lack of awareness regarding available applications [38,46].
- Patients’ self-perception of disease severity and satisfaction with current care [38].

 **Implementation process**

- Lack of systematic processes [37].
- Lack of time [40].
- Concerns regarding the quality of care [45].
- Financial risk assessment challenges [45].

**Facilitators**

 **Technology and Innovation**

- Free-of-cost accessibility [35].
- Simplified content for ease of understanding and convenient use (user-friendly, easy navigation, clear designs) [35,38,39].
- Provision of prompts and recommendations from health care providers [35].
- Standardized procedures provided for health care providers [35].
- Convenient use of the device in the home environment (vs in-person therapy) [34,39].
- Participants’ sense of control over the device/therapy [34].
- Remote monitoring by health care providers from patients’ perspectives [39].

 **Outer setting**

- Health care facilities’ commitment and patients’ demand [40].
- Disease management and reporting guidelines [40].
- Sustained electricity in clinics [47].

 **Inner setting**

- Direct provider-patient interaction [35,38].
- More senior physicians’ oversight and support to the DHT health care providers [35].
- DHT program integration with other services [35] or as an alternative offering within existing health care services [43].
- Teamwork, ongoing communication within the health care facility and assistance from the project team [40,43,45].
- Staff perceptions about the suitability of the program to address patient needs [40].
- Tangible rewards for patients [46].
- Establishing and adjusting workflows [43].
- Ongoing patient outreach and engagement [43].
- Anticipate and prepare for technology-related problems that may arise [43].
- Continuous training and support for the users to improve the skills to use the tool [47].
- Professional networking with experienced colleagues in DHT implementation [47].

 **Individuals**

- Perceived benefits and perceived usefulness of the DHT program from patients’ and providers’ perspectives [35,36].
- Patients’ needs, demand, and willingness to improve health [35,40].
- Perceived cost-benefits on patients and providers [35].
- Providers’ knowledge, capabilities, and confidence gained from training sessions [35].
- Providers’ perceived credibility from the top-down approach [35].

 **Implementation process**

- Effective leadership and collaboration with stakeholders [41].
- Service delivery tailored to local needs [41].
- Site feedback mechanism and evaluation of site readiness and uptake [41].
- Engaging local champions [41].

One study also raised concerns about the quality of care delivered via DHT [45]. The authors noted difficulties in ensuring that remote care maintained the same level of quality and effectiveness as in-person visits, potentially impacting patient satisfaction and outcomes [45].

##### User-Friendly Features and Convenience (Facilitators)

Conversely, several studies identified user-friendly features and convenience as key facilitators of DHT uptake. Free-of-cost access and simplified content, such as intuitive navigation and clear design, were among the most commonly cited enablers [35,38,39]. Furthermore, two studies highlighted the convenience of using DHTs in home settings as a significant advantage over traditional in-person therapy [34,39].

There were no discrepancies between qualitative and quantitative findings. Quantitative studies corroborated a few of the factors identified qualitatively.

### Outer Setting

#### Financial Barriers

Lack of sustainable funding for DHT programs and reimbursement of health care providers were among the most commonly reported barriers within this domain reported in three studies [35,37,45]. Gong et al [35] specifically highlighted the challenge of allocating funding in the absence of support from the county or higher authorities. According to James et al [37], limited funding led to staff unavailability during emergencies, such as device malfunctions or acute diabetes-related complications, outside regular office hours. Studies from Canada and Australia further underscored this issue, noting that reimbursement restrictions by the public systems (ie, Medicare) prevented many private practitioners from receiving payment for their services [37,45]. As a result, this situation hindered their willingness or ability to become more involved with DHT programs [37].

#### Resource Barriers

Another significant barrier identified was the lack of resources among patients. Insufficient internet connectivity, limited device ownership, and limited access to the necessary technology for the DHT program hindered its successful implementation [35,37,38,40]. Those findings were triangulated by barriers and a facilitator within the “Technology and Innovation” domain, highlighting that the cost to DHT devices or applications was a key factor in the program’s success [35,37,38]. In this domain, a study highlighted sustained electricity in rural facilities as a facilitator [47]. Additionally, commitments from health facilities and patient demand to address uncontrolled NCDs, such as diabetes, as well as existing guidelines for disease management, emerged as significant drivers of implementation [40].

### Inner Setting

Resource constraints and support within the health care environment emerged as key themes influencing the implementation and utilization of DHTs.

#### Resource Constraints

Across the findings of the selected studies, resource constraints were the most observed barrier. These constraints encompass challenges related to workload, staffing, a skilled workforce in technology, and infrastructure within implementing health facilities [35,37,40,47]. In one study, all clinics implementing a DHT innovation reported common challenges such as having a small number of staff and high rates of turnover [40]. This barrier was also echoed in other studies [37,47]. Moreover, the lack of expertise and staff skilled in IT, limited space, and supplies within the implementing clinics were found to be additional barriers within this domain [40,44,47]. Additional barriers are reported in Textbox 1 .

#### Support

Our synthesis identified support and collaboration within the health care environment as pivotal factors facilitating the effective implementation and utilization of DHTs across the selected studies. In their study, Kirkland et al [40] emphasized the importance of teamwork and communication within clinics for effective program delivery. Similarly, Livet et al [43] found that project teams’ flexibility, close engagement with DHT users (ie, health care providers and patients), and provision of assistance were crucial for success. Newell et al [45] echoed these findings, emphasizing the significance of open and honest communication and feedback between local providers, administrators, and DHT implementers. Two studies also highlighted the positive impact of periodic face-to-face interactions between patients and health care providers on the utilization and adoption of DHTs [35,38]. The integration of DHT programs with existing health care services or as an alternative offering was suggested to facilitate the integration and sustainability of the new DHT service in two studies [35,43]. Additional facilitators are presented in Textbox 1 .

### Individuals

Within this domain, we uncovered four themes: lack of motivation and reluctance to engage, facilitators like provider monitoring, age-related differences in adoption, and clinician expertise and confidence shaping uptake.

#### Lack of Motivation and Reluctance to Engage

The patient survey by Li et al [42] underscored several common barriers, such as a lack of motivation for food logging, limited time for exercise self-monitoring, and forgetfulness regarding blood glucose monitoring. Additionally, Jeffery et al [38] identified patient reluctance to take accountability for their health as a barrier to DHT usage. In the study by Li et al [42], major life events were also reported as barriers to engagement, including having relatives or close friends seriously ill or deceased (47%), immediate family illness or death (27%), and experiencing major financial difficulties (20%).

#### Impact of Health Care Provider Monitoring and Perceived Benefits

Patients’ perceptions of health care provider involvement played a significant role in DHT uptake. Jones et al [39] found that patients were more likely to use DHTs when they knew health care providers monitored them. These findings were in line with factors within the ’Technology/innovation’ domain, where features, such as remote monitoring and provider-generated prompts or recommendations, were seen as facilitators of successful implementation [35,37]. In contrast, Peng et al [46] reported that some patients chose not to use the DHT simply because their health care provider had not requested or encouraged its use. Perceived benefits and usefulness of the DHT program, viewed from the perspectives of both patients and providers, alongside patients’ needs, demands, and willingness to enhance their health, acted as facilitators within this domain [35,36,40]. This was further supported by quantitative findings, where perceived usefulness was positively associated with behavioral intention to use DHTs (*r*=0.585; *P*<.001), and behavioral intention was strongly associated with perceived ease of use (*r*=654; *P*<.001) [36]. Other facilitators reported by a single study included providers’ knowledge, capabilities, and confidence gained from training sessions, and providers’ perceived credibility from the top-down approach [35].

#### Clinician Expertise and Confidence Shaping Uptake

Across clinician-facing DHTs, individual providers’ experience, confidence, and beliefs about the value of the technology strongly influenced uptake. In rural telestroke, senior emergency department clinicians who were confident in their stroke or thrombolysis skills perceived little added value from telemedicine and were less likely to initiate a consult, whereas junior doctors sought specialist support, leading to uneven activation based on subjective need rather than standardized criteria [44]. Complementing this, James et al (2016) [37] reported that older (old-school) specialists held more negative views toward newer tools (apps), which hindered uptake, particularly in community-based health care settings where hands-on support for diabetes technologies was limited.

#### Age-Related Differences in Adoption

In one study, data stratified by age showed that participants aged 50‐66 years used technology extensively, while those aged 67‐87 years exhibited little to no use or intention to use technology, including email, text messaging, or computers, and declined assistance when offered [36]. Quantitative analysis further confirmed this relationship, demonstrating that older age was associated with lower behavioral intention to use DHTs (*r*=−0.047; *P*=.009) [36]. None of the qualitative data included demographic characteristics of patients or users, which could have been used to contrast or explain these findings.

### Implementation Process: Operational and Structural Barriers

The main barriers affecting the implementation process included a lack of systematic processes [37], insufficient time [40], and financial considerations [45]. James et al [37] reported that there were no established procedures in place to assess the benefits and risks of using the DHT device for each patient. Health care providers (ie, diabetes educators) were often challenged to decide whether a patient should use the technology, instead of using clear policies or professional guidance [37].

According to Kirkland et al [40], the top barrier was time, endorsed by 60% of clinics implementing DHT. Authors encountered difficulties in the initial stages of implementing the DHT, such as the financial impact on the rural site, the costs and benefits for patients, and the financial implications for the health care provider (ie, cardiology group) [45].

Leadership, collaboration, and tailored service delivery: effective leadership and collaboration with stakeholders, engagement of local champions, service delivery tailored to local needs, establishment of site feedback mechanisms, and evaluation of site readiness and uptake were identified as the primary facilitators of the implementation process in a single study [41].

## Discussion

### Principal Findings

This systematic review identified key barriers and facilitators shaping the implementation and uptake of DHTs for remote management of NCDs in rural settings. Across studies, key barriers included infrastructure and access constraints (eg, connectivity and technology availability), workforce limitations (eg, staffing shortages, workload, and limited IT expertise), and usability challenges. Facilitators included perceived benefits of DHTs, health care provider support and engagement, as well as effective teamwork and communication within clinical settings. By focusing on CVDs, hypertension, and diabetes, we aimed to provide a nuanced understanding of how these interventions function in resource-constrained environments. Applying CFIR enabled a structured organization of evidence, clarifying the practical, organizational, and contextual factors that shape success or failure.

Across studies, technical issues, such as software and hardware problems, poor navigation, and readability challenges, were common barriers to DHT uptake, alongside high costs and lack of personalized content [38,42,44,47]. These findings echo previous reviews identifying usability problems and technical complexity as major obstacles to DHT uptake more broadly [20,27]. However, unlike prior syntheses that combined rural and urban evidence [20,27], our rural-focused synthesis suggests these barriers are often amplified by limited infrastructure (eg, poor internet connectivity and limited access to devices or technology) [35,37,38,40] and constrained workforce capacity (eg, staffing shortages, turnover, and limited IT expertise) [35,37,40,44,47], which reduce the ability to provide training, troubleshooting, and sustained support.

Consistent with earlier evidence, facilitators included user-friendly design, simplified content, and the convenience of at-home use, reinforcing the importance of accessible design and perceived usefulness [35,38,39].

Patients’ limited access to internet connectivity, appropriate devices, and affordable technologies represented another major barrier [35,37,38,40]. Similar patterns have been reported in umbrella reviews, where infrastructure limitations and device availability featured prominently [20]. Yet earlier reviews rarely disaggregated findings by geography. By focusing specifically on rural contexts, our analysis highlights how these factors operate under more restrictive conditions, affecting both feasibility and sustainability.

Financial and structural barriers within the outer setting, including unsustainable funding, inadequate reimbursement models, and unreliable broadband or electricity, were also prominent [35,37,45]. Such barriers were particularly pronounced in rural settings, where infrastructure gaps, such as poor internet connectivity and unreliable electricity, remain pervasive [30,44,47]. This reflects global telehealth literature reporting similar barriers in diverse settings [27,28], but our findings emphasize that rural areas compounded inequities that elevate the risk of widening disparities if financial and infrastructural gaps remain unaddressed [48].

Resource constraints within the inner setting, limited staffing, workforce turnover, and lack of IT expertise were among the most prevalent barriers [35,37,40,47]. Although workforce limitations have been reported across urban health systems as well [49], rural environments experience more acute shortages and fewer opportunities for technical support or training [27]. Unlike complex urban systems, where challenges may relate to organizational coordination, rural challenges often seem to reflect absolute shortages in personnel or infrastructure.

The characteristics of individuals domain underscored the role of motivation, self-efficacy, and readiness for change [42]. Patterns observed in quantitative data, for example, low motivation for food logging, time limitations for self-monitoring, and forgetfulness, echoed themes in qualitative literature [38]. Age-related differences were also evident, with older adults (67‐87 y) showing minimal intention to use technology or accept support [36]. These findings mirror implementation literature emphasizing individual beliefs and self-efficacy as key determinants of uptake [32].

Provider influence further shaped uptake: in some studies, patients opted not to use DHT because their health care provider had not recommended them [46]. Conversely, provider endorsement significantly improved engagement [38]. Clinician perception similarly influenced implementation processes, with senior clinicians sometimes viewing telemedicine as redundant and time-consuming, whereas junior staff appreciated its value for support and shared decision-making [44]. These insights are consistent with wider implementation research highlighting clinician identity, perceived usefulness, and workflow compatibility as important determinants of technology uptake [50].

Process-related barriers, such as a lack of systematic procedures and insufficient time, also impeded implementation [37,40]. Meanwhile, facilitators, including strong leadership, teamwork, communication, presence of local champions, and tailored workflows, as well as periodic face-to-face interaction between providers and patients, reinforced the importance of adaptive strategies [35,38,40,41,45]. Evidence from previous reviews and qualitative studies confirms that collaboration and organizational supports are particularly critical in rural contexts where structural limitations require more compensatory mechanisms than in urban systems [51,52].

### Implications for Practice, Policy, and Research

#### Practice and implementation

Successful DHT uptake in rural health systems requires investment in organizational readiness: protected staff time, streamlined workflows, leadership engagement, local champions, and mechanisms for ongoing feedback and adaptation. Tailoring content and simplifying interfaces can reduce cognitive and time burden for both patients and providers, improving sustained use [35,37,38,40,41].

#### Policy and Financing

Persistent financial and infrastructure barriers point to the need for long-term, equity-oriented financing mechanisms. Priorities include sustainable reimbursement for remote monitoring, alignment of health care financing with digital infrastructure investment, and policies that recognize higher implementation and maintenance costs faced by rural health systems [27,28,48]. Equity-focused policies should also support broadband expansion and reliable electricity to avoid reinforcing disparities [30,44,47]. These directions are consistent with international digital health frameworks that stress infrastructure readiness, interoperability, and equity [53].

#### Research

Future research should prioritize generating context-specific evidence in underrepresented settings, particularly low- and middle-income countries. In addition, implementation-focused research on DHTs for the management of high-prevalent NCDs remains limited, underscoring the need for more comprehensive and context-sensitive evidence to inform implementation strategies.

### Integration of Quantitative and Qualitative Findings

A strength of this mixed methods systematic review is the triangulation of quantitative and qualitative evidence. Although quantitative data were limited, they complemented rather than contradicted the qualitative findings. For example, quantitative correlations between perceived usefulness and DHT uptake mirrored qualitative accounts from patients and providers who valued DHT benefits. Measures such as user satisfaction, perceived ease of use, perceived usefulness, adherence by patients and providers, and consistency are well established in digital health research and are useful for future work [54-56]. These metrics are commonly used to assess the adoption and sustainability of digital health and are considered essential for evaluating user experience and long-term engagement [55,56]. Incorporating such measures alongside qualitative insights would enhance triangulation, strengthen interpretation, and provide generalizable evidence to guide implementation strategies.

### Limitations

Several limitations should be considered when interpreting the findings of this review. Most studies included were conducted in high-income countries, with limited representation from low- and middle-income settings, which may affect the generalizability of findings to contexts with different health system capacities, resources, and infrastructure. This distribution was not restricted by the search strategy, which did not apply limits based on country income level, but rather reflects the availability of peer-reviewed evidence in this field, highlighting a relative lack of implementation-focused DHT studies in certain settings. It is also possible that relevant evidence, particularly from underrepresented contexts, exists in the gray literature, which was beyond the scope of this review.

While the included studies captured a broad range of perspectives and technologies, variability in study contexts, interventions, and populations may limit the direct transferability of specific barriers and facilitators across settings. Nevertheless, the consistency of key themes across studies suggests that many implementation factors may be broadly applicable, although they may require adaptation to local contexts.

This review was limited to English-language publications, which may have excluded relevant studies in other languages. Additionally, we did not include gray literature or conference proceedings, which may omit recent or emerging evidence. While this approach ensured a focus on high-quality, peer-reviewed manuscripts, it may have narrowed the scope of insights.

Finally, definitions of rural settings varied across included studies and were often based on authors’ descriptions rather than standardized criteria. In many cases, studies relied on general descriptors such as “rural” or “remote” without providing explicit classification frameworks. This lack of consistency limited the ability to systematically compare or categorize rural contexts across studies and may have introduced some heterogeneity in contextual classification. However, studies were carefully screened to ensure alignment with rural or nonurban contexts.

### Conclusion

DHTs for remote management of CVDs, hypertension, and diabetes in rural settings show promise but depend on addressing structural inequities, limited broadband, constrained workforce capacity, and fragile financing. Without targeted investments in infrastructure, reimbursement, and organizational readiness, digital tools risk entrenching rather than reducing disparities. An end-to-end approach—equity-focused design, context-responsive implementation, and sustained evaluation—can help ensure DHTs are usable, resilient, and aligned with rural health equity goals [53].

## Supplementary material

10.2196/93875 Multimedia Appendix 1 Search strategy.

10.2196/93875 Multimedia Appendix 2 Data extraction sheet.

10.2196/93875 Checklist 1 PRISMA 2020 checklist.

## Abbreviations

CFIR: Consolidated Framework for Implementation Research
CVD: cardiovascular disease
DHT: digital health technology
MMAT: Mixed Methods Appraisal Tool
NCD: noncommunicable disease
