# Supplementary material for: Barriers and Facilitators to Implementing Digital Health Technologies for Remote Management of NCDs in Rural Areas: Mixed Methods Systematic Review
Source: J Med Internet Res. 2026 Jul 31;28:e93875. doi: 10.2196/93875 (PMC13427061; doi:10.2196/93875)
Supplement: Multimedia Appendix 1 [file jmir-v28-e93875-s001.docx]

**MEDLINE (Ovid)**

**Search date:** February 15, 2024
**Database coverage:** 1946–present

1 telemedicine/ or remote consultation/ or telenursing/ or telerehabilitation/

2 ((mobile or virtual or digital) adj2 (health or medicine or therapeutic*)).ti,ab,kf.

3 (tele adj2 (consult* or monitoring)).ti,ab,kf.

4 (telehealth or telemedicine or ehealth or mhealth or telenursing or telecare or teleintervention or telethearpy).ti,ab,kf.

5 (remote adj2 consult*).ti,ab,kf.

6 (teleconsult* or telemonitoring).ti,ab,kf.

7 ((remote or virtual or tele) adj2 rehab*).ti,ab,kf.

8 Smartphone/

9 (smartphone* or (smart adj2 phone*)).ti,ab,kf.

10 or/1–9

11 rural.ti,ab,kf.

12 (rural adj2 (center* or centre* or service* or community or communities or population* or residence* or area* or patient*)).ti,ab,kf.

13 or/11–12

14 exp heart diseases/ or exp hypertension/ or exp myocardial ischemia/

15 stroke/ or exp brain infarction/ or exp hemorrhagic stroke/ or exp ischemic stroke/

16 diabetes mellitus/ or diabetes mellitus, type 1/ or diabetes mellitus, type 2/ or diabetes, gestational/

17 (diabetes or diabetic).ti,ab,kf.

18 ((heart or cardiac) adj2 (disease* or condition* or syndrome* or disorder* or failure)).ti,ab,kf.

19 or/14–18

20 10 and 13 and 19

**Embase (Embase Classic + Embase)**

**Search date:** February 15, 2024
**Database coverage:** 1947–present

1 telemedicine/ or remote consultation/ or telenursing/ or telerehabilitation.mp.

2 ((mobile or virtual or digital) adj2 (health or medicine or therapeutic*)).ti,ab.

3 (tele adj2 (consult* or monitoring)).ti,ab.

4 (telehealth or telemedicine or ehealth or mhealth or telenursing or telecare or teleintervention or telethearpy).ti,ab.

5 (remote adj2 consult*).ti,ab.

6 (teleconsult* or telemonitoring).ti,ab.

7 ((remote or virtual or tele) adj2 rehab*).ti,ab.

8 Smartphone/

9 (smartphone* or (smart adj2 phone*)).ti,ab.

10 or/1–9

11 rural health services/ or rural nursing/

12 Rural Population/

13 rural.ti,ab.

14 (rural adj2 (center* or centre* or service* or community or communities or population* or residence* or area* or patient*)).ti,ab.

15 or/11–14

16 exp heart diseases/ or exp hypertension/ or exp myocardial ischemia/

17 stroke/ or exp brain infarction/ or exp hemorrhagic stroke/ or exp ischemic stroke/

18 diabetes mellitus/ or diabetes mellitus, type 1/ or diabetes mellitus, type 2/ or diabetes, gestational/

19 (diabetes or diabetic).ti,ab.

20 ((heart or cardiac) adj2 (disease* or condition* or syndrome* or disorder* or failure)).ti,ab.

21 or/16–20

22 10 and 15 and 21

**Database:** CINAHL Complete (EBSCOhost)
**Coverage:** 1981–present
**Search date:** January 20, 2024

S1 TI (telemedicine OR "remote consultation" OR telenursing OR telerehabilitation)

OR AB (telemedicine OR "remote consultation" OR telenursing OR telerehabilitation)

S2 TI ((mobile OR virtual OR digital) N2 (health OR medicine OR therapeutic*))

OR AB ((mobile OR virtual OR digital) N2 (health OR medicine OR therapeutic*))

S3 TI ((tele N2 (consult* OR monitoring)))

OR AB ((tele N2 (consult* OR monitoring)))

S4 TI (telehealth OR telemedicine OR ehealth OR mhealth OR telenursing OR telecare OR teleintervention OR teletherapy)

OR AB (telehealth OR telemedicine OR ehealth OR mhealth OR telenursing OR telecare OR teleintervention OR teletherapy)

S5 TI (remote N2 consult*) OR AB (remote N2 consult*)

S6 TI (teleconsult* OR telemonitoring) OR AB (teleconsult* OR telemonitoring)

S7 TI ((remote OR virtual OR tele) N2 rehab*) OR AB ((remote OR virtual OR tele) N2 rehab*)

S8 TI Smartphone/ OR AB Smartphone/

S9 TI (smartphone* OR (smart N2 phone*)) OR AB (smartphone* OR (smart N2 phone*))

S10 S1 OR S2 OR S3 OR S4 OR S5 OR S6 OR S7 OR S8 OR S9 [telehealth]

S11 TI ("rural health services" OR "rural nursing")

OR AB ("rural health services" OR "rural nursing")

S12 TI "Rural Population" OR AB "Rural Population"

S13 TI rural OR AB rural

S14 TI ((rural N2 (center* OR centre* OR service* OR community OR communities OR population* OR residence* OR area* OR patient*)))

OR AB ((rural N2 (center* OR centre* OR service* OR community OR communities OR population* OR residence* OR area* OR patient*)))

S15 S11 OR S12 OR S13 OR S14 [rural]

S16 TI ("heart diseases" OR hypertension OR "myocardial ischemia")

OR AB ("heart diseases" OR hypertension OR "myocardial ischemia")

S17 TI (stroke OR "brain infarction" OR "hemorrhagic stroke" OR "ischemic stroke")

OR AB (stroke OR "brain infarction" OR "hemorrhagic stroke" OR "ischemic stroke")

S18 TI ("diabetes mellitus" OR "diabetes mellitus, type 1" OR "diabetes mellitus, type 2" OR "diabetes, gestational")

OR AB ("diabetes mellitus" OR "diabetes mellitus, type 1" OR "diabetes mellitus, type 2" OR "diabetes, gestational")

S19 TI (diabetes OR diabetic) OR AB (diabetes OR diabetic)

S20 TI ((heart OR cardiac) N2 (disease* OR condition* OR syndrome* OR disorder* OR failure))

OR AB ((heart OR cardiac) N2 (disease* OR condition* OR syndrome* OR disorder* OR failure))

S21 S16 OR S17 OR S18 OR S19 OR S20 [chronic]

S22 S10 AND S15 AND S21
